# Increased Circulating Advanced Oxidation Protein Products and High-Sensitive Troponin T in Cirrhotic Patients with Chronic Hepatitis C: A Preliminary Report

**DOI:** 10.1155/2015/786570

**Published:** 2015-11-19

**Authors:** Jolanta Zuwala-Jagiello, Eugenia Murawska-Cialowicz, Monika Pazgan-Simon

**Affiliations:** ^1^Department of Pharmaceutical Biochemistry, Wroclaw Medical University, 211 Borowska Street, 50556 Wroclaw, Poland; ^2^Department of Physiology and Biochemistry, University of Physical Education, 35 Paderewskiego Avenue, 51612 Wroclaw, Poland; ^3^Clinic of Infectious Diseases, Liver Diseases and Acquired Immune Deficiency, Wroclaw Medical University, 5 Koszarowa Street, 51149 Wroclaw, Poland

## Abstract

*Aim*. To investigate the relationship between advanced oxidation protein products (AOPPs) and myocardial injury by comparing the selected biomarker for detecting myocardial injury [high-sensitive troponin T (hs-TnT)] in patients with chronic HCV infection.* Methods and Results*. Eighty-eight patients with cirrhosis and 40 healthy control subjects were enrolled in the study. Circulating levels of AOPPs-albumin (the ratio of AOPPs to albumin content), hs-TnT, tumor necrosis factor *α* (TNF-*α*), and high-sensitivity C-reactive protein (hs-CRP) were assessed. Compared with healthy controls, the cirrhotic patients with chronic HCV infection had higher levels of AOPPs-albumin, which were associated with increased hs-TnT. When the presence of ascites was considered, the plasma levels of AOPPs-albumin were higher, as well as TNF-*α*. AOPPs-albumin positively correlated with hs-TnT level in all cirrhotic patients with chronic HCV infection and this correlation was stronger in decompensated cirrhotic patients. In multivariate logistic regression analysis, the independent factors associated with the presence of ascites were high AOPPs-albumin levels and elevated hs-TnT levels.* Conclusion*. The simultaneous monitoring of plasma AOPPs and hs-TnT can be helpful for the alterations in myocardial function control in cirrhotic patients with chronic HCV infection.

## 1. Introduction

Patients with hepatitis C virus (HCV) infection who are chronically infected may go on to develop cirrhosis and hepatocellular carcinoma. Most authors favor the opinion that HCV infection is frequently associated with myocarditis and cardiomyopathy [1, 2]. In fact, it has been reported that HCV is replicated in myocardial tissue of patients with myocarditis; thus, HCV infection may contribute to the development of this form of myocarditis [2]. The severity of myocarditis associated with HCV infection is highly variable [2, 3]. In HCV heart failure, most patients develop chronic inflammation of the myocardium and later dilated cardiomyopathy attributable to necrosis and loss of myocytes. However, because myocytes do not replicate, the proliferative stimuli induced by HCV infection may promote myocyte hypertrophy and hypertrophic cardiomyopathy [4]. The presence of left ventricular hypertrophy and diastolic dysfunction in patients with chronic hepatitis C (CHC) during the precirrhotic stage also suggests a possible role of HCV in this structural abnormality of the heart [5]. Furthermore, the functional and structural cardiac abnormalities are present in the majority of CHC patients with moderately or severely advanced failure (Child-Pugh stage B or C) [6, 7]. Generally, myocardial injury worsens with the progression of the underlying liver failure. Therefore, the use of reliable biochemical markers for the detection of myocardial damage is essential in these patients.

Troponin T (TnT) and troponin I (TnI) are present in cardiac muscles that regulate inflammatory processes and play a leading role in myocardial hypertrophy associated with heart failure and left ventricular hypertrophy. Beyond the potential to screen for structural cardiac abnormalities, recent data suggest that cardiac troponins are valuable for screening asymptomatic individuals to prevalent subclinical cardiovascular disease [8, 9]. The high prevalence of elevated serum levels of cardiac troponins in heart failure raises the question of what mechanism of cardiomyocyte damage results in troponin release in heart failure. Proposed mechanisms include inflammatory cytokines, oxidative stress, and apoptosis [10, 11]. However, relationships between apoptosis and troponin plasma concentrations remain to be shown. Because cardiovascular disease begins early in the course of liver failure, differentiation of sources of elevated hs-TnT levels in cirrhotic patients is difficult. Nevertheless, the hs-TnT elevation is an independent predictor for adverse events [12]. Everett et al. [13] speculated that the very low, but detectable hs-TnT levels, may reflect a normal biological process of myocyte turnover. Even though the regeneration of myocytes may contribute to an increase in the muscle mass of the myocardium, a gradual decrease <50% of cardiomyocytes is exchanged during a normal lifespan [14]. Thus, it is not surprising that higher hs-TnT levels predicted a worse prognosis regardless of the conditions in cardiac or noncardiac diseases.

Oxidative stress leads to formation of glycoxidation products, including advanced oxidation protein products (AOPPs) and advanced glycation end products (AGEs). AOPPs can be formed* in vitro* by the exposure of serum albumin to hypochlorous acid.* In vivo*, plasma AOPPs are mainly carried by albumin, and their concentrations are closely correlated with the levels of dityrosine. The receptor for advanced glycation end products (RAGE) is a signal transduction receptor that binds both AGEs and AOPPs. RAGE is expressed by various cell types, including endothelial cells, smooth muscle cells, renal cells, and cardiomyocytes [15, 16]. Binding of AOPPs to the RAGE results in the generation of reactive oxygen species. Thus, AOPPs, formed as a result of oxidative stress, induce ROS generation* via* NADPH oxidase and can perpetuate oxidative stress conditions. Of note,* RAGE* is also an oxidative stress-responsive gene. The* RAGE* gene promoter contains several transcription factor-binding sites, including nuclear transcription factor [kappa]B (NF-*κ*B) [17]. In cardiomyocytes, activation of NF-*κ*B has been generally shown to activate cell survival pathways [18]. However, AOPPs suppress cell proliferation* via* the activation of NF-*κ*B and induce cardiomyocyte death* via *RAGE [16, 19]. Valente et al. [16] first elucidated the role of AOPPs in proapoptotic signaling in cardiomyocytes. Of note, apoptosis of cardiomyocytes is a prerequisite for myocardial hypertrophy and heart failure-related remodeling. Systemic levels of AOPPs are increased in diverse chronic oxidative conditions, including diabetes mellitus, chronic kidney, and contribute to cardiac diseases [20, 21]. Since cardiovascular diseases are the major contributors of morbidity and mortality, it is possible that increased AOPPs-mediated cardiomyocyte death might perpetuate myocardial injury in CHC patients with moderately or severely advanced liver failure.

The aims of this study were to evaluate preliminarily the plasma levels of both AOPPs and hs-TnT of patients with chronic HCV infection for comparison with cirrhotic patients and to determine what factors significantly influence the hs-TnT level of CHC patients.

## 2. Patients and Methods

### 2.1. Patients

This study was performed in 120 patients with chronic HCV infection admitted to the Clinic of Infectious Diseases, Liver Diseases and Acquired Immune Deficiency for evaluation. The patients with chronic HCV infection were divided into two groups in function of the presence (88 patients) or the absence (32 patients) of cirrhosis. A total of 88 cirrhotic patients were included in the study.

Fifty-three were male and 35 were female, and they were aged 21–74 years (median age 56 years). The control group consisted of healthy blood donors with normal aminotransferases, normal blood counts, and negative markers for virus hepatitis and HIV (23 males/17 females, median age: 55 years). Blood samples were collected in the Department of Physiology and Biochemistry, University of Physical Education in Wroclaw. Clinical and biochemical characteristics of the study group are reported in detail in Table 1.

Inclusion criteria were histological or clinical diagnosis of cirrhosis, no evidence of metabolic, toxic, or autoimmune liver disease, and at least 1 year of alcohol abstinence. Diagnosis of cirrhosis was established according histological criteria when liver biopsy was performed, or by the combination of clinical, biochemical, and ultrasound imaging data (presence of irregular margins on ultrasound, portal hypertension with laboratory evidence of chronic liver disease), consistent with such a diagnosis. Patients were grouped according to Child-Pugh classification. Three biochemical variables [serum albumin, bilirubin, and prothrombin time (international normalized ratio, INR)] in addition to the presence or absence of ascites determine the Child-Pugh score. At the time of the study, no Child-Pugh A patients showed clinical features of decompensated liver cirrhosis (ascites or hepatic encephalopathy). At enrollment, esophageal varices were detected by endoscopy in 53% of patients; ascites and hepatic encephalopathy grade I were present by physical examination in 53 (60%) and 23 (26%) patients, respectively. Presence of ascites was assessed by ultrasonography. Bacterial infection was ruled out by clinical history, physical examination, differential and total white blood cell count, analysis and culture of urine, thorax X-ray, and culture and white blood cell count of ascetic fluid in patients with ascites.

Exclusion criteria were concurrent use of antioxidant drugs, coexisting diseases like chronic kidney disease, diabetes mellitus, cardiovascular disease, cardiac decompensation, and hepatocellular carcinoma, gastrointestinal bleeding, bacterial infection, and blood transfusion within previous two weeks. Patients received no diuretic, antibiotic, vasoactive drug (nitrates, *β*-blockers) and lactulose or lactitol therapy during the eight days before inclusion in the study.

After 2 h of bed rest, blood pressure was determined with an automatic digital sphygmomanometer and blood samples were collected in ice-cooled, ethylenediaminetetraacetic acid- (EDTA-) containing tubes for the determination of plasma renin activity, antidiuretic hormone, and plasma AOPPs or hs-TnT in tubes with no additive for routine biochemical study and aldosterone and inflammatory markers concentrations. All samples were separated immediately by centrifugation at 4°C and stored at −80°C until further analysis.

The consent of the Bioethics Committee of the Wroclaw Medical University was obtained and all patients were informed about the character of the analyses made. Studies were conducted in compliance with the ethical standards formulated in the Helsinki Declaration of 1975 (revised in 1983).

### 2.2. Determination of Circulating AOPPs

Determination of AOPPs was based on spectrophotometric detection according to Šebeková et al. [22]. Two hundred microliters of plasma diluted 1 : 5 in 20 mM phosphate buffer pH 7.4 containing 0.9% sodium chloride (PBS), or chloramine-T standard solutions (0 to 100 *μ*mol/L), was placed in each well of a 96-well microtiter plate (Becton Dickinson Labware, Lincoln Park, NJ, USA), followed by 20 *μ*L of 10% acetic acid. Ten microliters of 1.16 M potassium iodide (Sigma) were then added, followed by 20 *μ*L of 10% acetic acid. The absorbance of the reaction mixture was immediately read at 340 nm in a microplate reader against a blank containing 200 *μ*L of PBS, 10 *μ*L of KI, and 20 *μ*L of 10% acetic acid. The chloramine-T absorbance at 340 nm was linear within the range of 0 to 100 *μ*mol/L. The ratio of AOPPs concentration to albumin level (AOPPs-albumin) was expressed in micromoles of AOPPs per gram of albumin (*μ*mol/g). The ratio of AOPPs to albumin content allows the evaluation of whether the proportion of oxidatively modified albumin is altered. Coefficient of variation (CV) served as an indicator of precision. Intraday and interday CV values were <10%.

### 2.3. Laboratory Determinations

*Biochemical parameters* were measured using routine laboratory methods. Serum high-sensitivity C-reactive protein (hs-CRP) level was determined with a high-sensitivity nephelometric method using the Beckman IMMAGE Immunochemistry System (Beckman Instruments, Fullerton, CA), which has a minimum level of detection of 0.2 mg/L. Serum levels of TNF-*α* were assayed with enzyme-linked immunosorbent assay (ELISA) kits (R&D Systems Inc., Minneapolis, MN, USA) according to the manufacturer's instructions. The minimum levels of detection were 1.6 pg/mL for TNF-*α*. The intra- and interassay coefficients of variation for measurements of hs-CRP and TNF-*α* were 2.7% and 5.0%, respectively, and 3.0% and 6.9%, respectively.

High-sensitive troponin T was measured using Cobas Troponin T hs (highly sensitive) STAT (short turn-around time) (Roche Diagnostics). The assay working range is reported as 3–10 000 ng/L, with an interassay CV, according to the manufacturer, of 3.1% at 24 ng/L and 1.3% at 300 ng/L. The lower limit of quantification is 13 ng/L, the limit of detection is 5 ng/L, and the limit of blank is 3 ng/L, as listed by the manufacturer.

### 2.4. Statistical Analysis

Continuous variables are expressed as median [interquartile range (IQR)] and categorical variables as number (percentage). Frequency data were compared using the *χ* ^2^ test or Fischer's exact test when necessary. Because many of the variables analyzed did not have a normal distribution as determined by the Kolmogorov-Smirnov test, nonparametric tests were used for comparison of data. The Mann-Whitney *U*-test and the Kruskal-Wallis test were used to analyze differences among two or more groups, respectively. Multivariate analysis by conditional logistical regression with a forward stepwise method was performed to find independent variables associated with the presence of ascites. Regression analysis to determine significant correlations among different parameters was performed using the Spearman correlation coefficient. Statistical significance was established at *P* < 0.05.

## 3. Results

### 3.1. AOPPs-Albumin and hs-TnT Plasma Levels in Patients with Liver Cirrhosis and Healthy Controls

We analyzed 88 cirrhotic patients (53 males/35 females, median age: 56 years, range: 21–74 years) with chronic HCV infection. AOPPs-albumin plasma concentrations were significantly higher in CHC patients without ascites than in healthy controls (controls median 1.7 *μ*mol/g, IQR 0.8–2.7 *μ*mol/g) (*P* < 0.05, Table 1). In healthy controls, the plasma AOPPs-albumin concentrations were similar to those in control groups in other studies [22]. AOPPs-albumin plasma concentration was significantly higher in cirrhotic patients (*n* = 88; median 2.4 *μ*mol/g, IQR 1.3–5.2 *μ*mol/g) compared to CHC patients without cirrhosis (*n* = 32; median 2.1 *μ*mol/g, IQR 0.9–3.0 *μ*mol/g) (*P* < 0.05, Table 1).

The distribution of the stages of liver cirrhosis as defined according to the Child-Pugh score, and measurements of AOPPs-albumin, and hs-TnT concentrations is presented in Table 2. Patients with Child-Pugh class C exhibited significantly higher plasma concentrations of AOPPs-albumin than patients with Child-Pugh class A and controls (*P* < 0.05, *P* < 0.01, resp.). There was a significant difference between Child-Pugh B cirrhotic patients and control subjects with respect to AOPPs-albumin level (Table 2). In CHC patients without cirrhosis, hs-TnT had a median value of 5.6 ng/L (IQR 3.0–7.1 ng/L) (Table 1). Plasma hs-TnT concentrations were higher in Child-Pugh A to Child-Pugh C cirrhotic patients (*n* = 88; median 7.9 ng/L, IQR 3.0–18.5 ng/L) than in patients without cirrhosis, but this difference was not statistically significant (Table 1). hs-TnT plasma concentration was significantly higher in patients with Child-Pugh class C cirrhosis compared to patients with Child-Pugh class A cirrhosis (*P* < 0.05, Table 2). There was statistically significant correlation between hs-TnT levels and the Child-Pugh score in cirrhotic patients (*r* = 0.25, *P* < 0.01, Table 3). AOPPs-albumin positively correlated with the hs-TnT, both when the whole group of cirrhotic patients was evaluated (*r* = 0.28, *P* < 0.05) and when correlation analysis was limited to patients with ascites (*r* = 0.35, *P* < 0.01).

According to an analysis relating AOPPs-albumin and hs-TnT level to the presence of complications of cirrhosis for patients as indicated by the presence of esophageal varices, hyperbilirubinemia, and prolonged INR, there were no significant differences. However, in CHC patients with cirrhosis, AOPPs-albumin correlated inversely with the serum albumin (*r* = −0.38, *P* < 0.05). Significant correlations between AOPPs-albumin and hs-TnT level and MELD scores (*r* = 0.43, *P* < 0.001; *r* = 0.31, *P* < 0.001, resp.) were observed among the cirrhotic patients belonging to all three Child-Pugh classes. In the study group, no significant correlations were also observed between AOPPs-albumin and hs-TnT level and biochemical markers of liver injury (not reported in detail).

We assessed the levels of several inflammatory markers and their association with the levels of AOPPs-albumin and hs-TnT. Serum high-sensitivity C-reactive protein (hs-CRP) levels were significantly elevated in cirrhotic patients (Table 2). Serum TNF-*α* levels were higher in the Child-Pugh class C cirrhosis than in the Child-Pugh class A cirrhosis (*P* < 0.05, Table 2). Moreover, TNF-*α* concentrations were positively correlated with Child-Pugh score in cirrhotic patients (*r* = 0.31, *P* < 0.05). There was no statistically significant correlation between AOPPs-albumin and hs-TnT level and hs-CRP or TNF-*α* levels in all liver cirrhotic patients (data not shown).

### 3.2. Clinical and Biochemical Characteristics of Patients with Liver Cirrhosis according to the Presence of Ascites

The biochemical and clinical characteristics of cirrhotic patients both with and without ascites are shown in Table 3. Distribution of sex was similar among groups. By design, creatinine, aldosterone levels, and plasma renin activity were higher in cirrhotic patients with ascites than in patients without ascites. However, similar values of antidiuretic hormone were detected in all patients grouped according to the presence of ascites. CHC patients with ascites had significantly lower values of mean arterial pressure (MAP) when compared with CHC patients without ascites (*P* < 0.05).

AOPPs-albumin levels were significantly different between nonascites and CHC patients with ascites (*n* = 53; median 3.6 *μ*mol/g, IQR 1.9–5.2 *μ*mol/g) (Table 3). The association study revealed negative correlation between AOPPs-albumin levels and MAP in all cirrhotic patients (*r* = −0.27, *P* < 0.05).

Plasma hs-TnT levels in ascites group were significantly increased compared with those in nonascites group (*P* < 0.01, Table 3). Additionally, hs-TnT and AOPPs-albumin level in refractory ascites (*n* = 11) was significantly increased compared with the nonascites group [median 12.9 ng/L (IQR 8.4–18.5 ng/L), median 4.3 *μ*mol/g (IQR 3.3–5.2 *μ*mol/g), resp.]; *P* < 0.001.

The median TNF-*α* levels were higher in CHC patients with ascites than patients without ascites (55.0 pg/mL versus 32.3 pg/mL, *P* < 0.01) (Table 3). There was also no significant difference between serum TNF-*α* levels in patients with the elevated levels of circulating AOPPs-albumin and levels in patients with the elevated levels of hs-TnT (data not shown).

hs-CRP plasma levels were increased in the nonascites and ascites group, with higher levels in the latter (Table 2). Serum hs-CRP levels were variable and no correlation with the hs-TnT was found (*P* > 0.05).

### 3.3. Multiple Regression Analysis

Based on stepwise multiple logistic regression analysis of factors (Child-Pugh classification, MELD score, bilirubin, albumin, creatinine, low serum sodium concentration, INR, MAP AOPPs-albumin, hs-TnT, and TNF-*α*), plasma AOPPs-albumin level [odds ratio (OR) = 0.19, 95% confidence interval (CI) = 0.21–0.44, *P* < 0.001], hs-TnT level [odds ratio (OR) = 0.11, 95% confidence interval (CI) = 0.17–0.61, *P* < 0.001], and creatinine level (OR = 0.64, 95% CI = 0.02–0.03, *P* < 0.001) were found to be independent predictors of ascites (Table 4).

## 4. Discussion

In the present study, we investigated the relationship between AOPPs-albumin and myocardial injury by comparing the selected biomarker for detecting cardiac injury (hs-TnT) in patients with chronic HCV infection showing high levels of AOPPs-albumin in the presence of high serum TNF-*α* levels. The main findings are as follows. (1) Compared with healthy controls, the cirrhotic patients had higher levels of AOPPs-albumin, which were associated with increased hs-TnT. (2) When the presence of ascites was considered, the plasma levels of AOPPs-albumin were higher, as well as TNF-*α*. (3) AOPPs-albumin positively correlated with hs-TnT level in all cirrhotic patients with chronic HCV infection and this correlation was stronger in decompensated cirrhotic patients. (4) In multivariate logistic regression analysis, the independent factors associated with the presence of ascites were high AOPPs levels and elevated hs-TnT levels.

The associations between HCV infection and cardiovascular diseases are supported by a robust body of evidence [2, 4, 23]. It is therefore possible that myocardial inflammation or virus persistence, or both, may cause an asymmetrical septal hypertrophy enabling the occurrence of hypertrophic cardiomyopathy [24]. Myocardial hypertrophy and remodeling are pathological features of many cardiac diseases, with the underlying causes including cirrhotic cardiomyopathy in patients with chronic HCV infection. This syndrome designates a cardiac dysfunction that includes the macroscopic structural changes, systolic and/or diastolic dysfunction, and electrophysiological changes [6]. In the present study, elevated plasma concentrations of hs-TnT were associated with increases in the concentrations of AOPPs-albumin and TNF-*α* in cirrhotic patients with chronic HCV infection. Although these biochemical substances are known to be elevated in patients with heart failure whose prognosis is poor [25–27], there have been several reports suggesting they induce cardiomyocyte apoptosis, a critical component in the pathogenesis of heart failure [28]. Elevated hs-TnT serum levels were associated with the severity of hypertrophic cardiomyopathy, indicating that the hs-TnT levels could be a reliable indicator of subclinical ongoing myocyte damage [10]. In fact, high-sensitive assay methods for cardiac troponins are nowadays under an intense development since cardiac troponins are biomarkers of cardiomyocyte apoptosis [11], remodelling processes, or increased physiological cell turnover occurring in different etiologic origins of cardiac injury [10]. Very recently, Valente et al. [16] first elucidated the role of AOPPs in proapoptotic signaling in cardiomyocytes. These studies concluded that AOPPs-modified mouse serum albumin induces cell death in both neonatal and adult mouse cardiomyocytes, and this effect is mediated* via* RAGE. AOPPs-induced Nox2/Rac1-dependent superoxide generation and increased TRAF3 interacting protein 2 (TRAF3IP2) expression and TRAF3IP2-dependent c-Jun N-terminal kinase (JNK) activation. Further, AOPPs-MSA induced mitochondrial Bax translocation and release of cytochrome c into cytoplasm. Moreover, AOPPs-MSA suppressed antiapoptotic Bcl-2 and Bcl-xL expression. Knockdown of the adapter protein TRAF3IP2 blunted AOPPs-induced apoptosis in both neonatal and adult cardiomyocytes [16]. The ability of the appropriate stimulus to drive cardiomyocytes into apoptosis indicated that these cells were primed for apoptosis and were susceptible to clinically relevant apoptotic triggers, such as AOPPs. Our preliminary results are the first to show a correlation between marker of cardiomyocyte injury (hs-TnT) and circulating AOPPs-albumin in CHC patients with cirrhosis. These results further demonstrate that the myocardial troponin T release was dependent on the AOPPs-albumin elevation because, in cirrhotic patients with normal AOPPs-albumin levels, detectable hs-TnT concentration was not significantly elevated in comparison with patients with chronic HCV infection with normal AOPPs-albumin levels (not shown). Accordingly, there are reasons to believe that the cirrhotic myocardium may be more vulnerable to the deleterious effects of elevated AOPPs.

Most cirrhotic patients remain asymptomatic until the occurrence of decompensation characterized by ascites. The changes in cardiac function during cirrhotic cardiomyopathy are more evident in decompensated cirrhosis, and TNF-*α* is key factor in the signaling pathways regulating myocardial dysfunction. Studies, in fact, show that TNF-*α* induces apoptosis in cardiomyocytes [29, 30], suppresses cardiac contractility [31, 32], and provokes myocardial hypertrophy [33, 34]. Very recently, Che et al. [35] reported the possible association between diastolic dysfunction and inflammation reflected by serum TNF-*α* levels in patients with HCV infection. The other study assessing the role of NF-*κ*B in mediating systolic dysfunction in cirrhosis showed an improvement of diastolic relaxation in cardiomyocytes when its inhibitors blocked NF-*κ*B activity, with reduction in TNF-*α* expression [36]. This suggests that an inflammatory milieu, with increased TNF-*α* levels, may also be partly responsible for the diastolic dysfunction in cirrhosis, but the exact mechanism with which TNF-*α* affects diastolic dysfunction has not been elucidated. Increased TNF-*α* level, along with hs-TnT levels in cirrhotic patients, especially higher values in patients with ascites, was observed in the present study. Since TNF-*α* is associated with diastolic dysfunction, it is likely to affect the progress of myocardial injury in patients with advanced liver failure. However, in the present study, there was no significant correlation between serum TNF-*α* levels and increased plasma levels of hs-TnT in cirrhotic patients. These data suggest that, although TNF-*α* might contribute to the heart failure in CHC patients with cirrhosis, especially with advanced disease, other factors acting through different pathways probably exist.

Elevated levels of both AOPPs-albumin and hs-TnT were detected in the early stages of liver dysfunction: plasma concentrations were increased in patients in Child-Pugh class A, with higher values found in those in class B or C. Furthermore, circulating levels of both AOPPs-albumin and hs-TnT were associated with disease severity by significant relations to the MELD score (*r* = 0.43, *P* < 0.001; *r* = 0.31, *P* < 0.001, resp.). This is in keeping with previously reported data [12, 37]. Our results also showed that hs-TnT levels in all cirrhotic patients were independently associated with the presence of ascites and showed significant correlations with AOPPs levels. Additionally, increased circulating AOPPs-albumin level, along with hs-TnT levels in decompensated cirrhotic patients, especially higher values in refractory ascites group, were observed in this study. While our data must be interpreted cautiously because they are of a cross-sectional nature, our findings are consistent with those of Wiese et al. showing a relation between cardiac dysfunction and development of refractory ascites [12]. Finally, the values of both AOPPs-albumin and hs-TnT in CHC patients with a low Child-Pugh score and absence of ascites suggest that AOPPs might have a role in the late stages of cirrhosis by aggravating the already initiated cardiac dysfunction.

Analysis of plasma hs-TnT levels in cirrhotic patients has to deal with the presence of impaired renal function as a possible confounder. It has been suggested that especially in subjects with impaired renal function TnT may be falsely elevated. Reduced clearance of troponin molecules or their fragments by dysfunctional kidneys may explain persistently elevated circulating concentrations of troponin in patients with chronic heart failure [38], but this hypothesis is controversial [39]. Rather, elevated troponin levels most likely indicate myocardial injury [39–41]. It has recently been shown that renal excretion of TnT is significantly impaired in patients with alcohol-related cirrhosis, and this may at least in part explain elevated plasma levels in these patients [12]. In contrast, in the present study, elevated serum levels of creatinine were not significantly correlated with increased levels of hs-TnT (not shown). Our data agree with other studies [40, 42] showing that the clearance of cardiac troponins is not changed by renal failure. The results of the measurements of troponins renal clearance might depend on which of the epitopes that are detected by individual assays and by the degradation of troponin.

Our study has a number of limitations that merit consideration. First, our sample size is relatively small, and a larger cohort of cirrhotic patients with HCV infection will need to be examined to confirm our findings. Second, interpreting the present data is limited by the small number of the patients studied, resulting in a limited statistical power. Third, the limit of the present study was the unavailability of brain natriuretic peptide and probrain natriuretic peptide, which are also recognized to be important in the evaluation of diastolic dysfunction. Lastly, we did not perform serial measurements and only focused on baseline values. Accordingly, our cross-sectional study design does not permit any conclusions on causality.

In conclusion, results from this study show that elevated levels of both AOPPs and hs-TnT are common in cirrhotic patients with HCV infection. Moreover, the present results provide new evidence for an association between plasma levels of AOPPs and hs-TnT, a specific marker of myocardial injury, in patients with decompensated cirrhosis. Although it does not prove causal relationship, it might strengthen the hypothesis that AOPPs are associated with cardiac disease in cirrhotic patients with HCV infection. Further investigation will be needed to elucidate the mechanisms underlying the regulation of advanced oxidation protein products, hs-TnT and other cardiovascular markers in cirrhotic patients with hepatitis C virus (HCV) infection.

## Figures and Tables

**Table 1:** Clinical and biochemical characteristics of the study subjects.

	Healthy controls	Noncirrhotic patients	Cirrhotic patients
(*n*)	40	32	88
Male : female ratio	23 : 17	7 : 25	53 : 35
Age (years)	55 (29–56)	56 (38–69)	56 (21–74)
Ascites *n* (%)	—	—	53 (73)
Esophageal varices *n* (%)	—	—	47 (53)
Albumin (g/L)	45 (36–57)	37^*∗*^ (29–49)	30^*∗*^ (16–45)
ALT (U/L)	24 (20–28)	28 (24–33)	47^*∗∗*^ (16–79)
AST (U/L)	27 (23–30)	41 (19–64)	79^*∗∗*^ (19–150)
Bilirubin (mg/dL)	0.7 (0.6–0.9)	0.92 (0.90–0.95)	1.6^*∗*^ (1.0–3.6)
*γ*GT (U/L)	26 (25–28)	48 (41–56)	92^*∗∗*^ (78–106)
Creatinine (mg/dL)	0.8 (0.7–1.2)	0.96 (0.9–1.2)	1.2^*∗*^ (0.7–2.4)
Serum sodium (mmol/L)	140 (138–141)	136 (129–138)	130^*∗∗*^ (129–142)
Mean arterial blood pressure (mmg/Hg)	—	—	83 (76–93)
hs-TnT (ng/L)	—	5.6 (3.0–7.1)	7.9 (3.0–18.5)
AOPPs-albumin (*μ*mol/g)	1.7 (0.8–2.7)	2.1^*∗*^ (0.9–3.0)	2.4^*∗*^ (1.3–5.2)

Continuous variables are expressed as median (interquartile range, IQR) and categorical variables as number (percentage). Statistical significance: ^*∗*^ *P* < 0.05; ^*∗∗*^ *P* < 0.01  *versus* healthy controls. ALT: alanine aminotransferase; AOPPs: advanced oxidation protein products; AST: aspartate aminotransferase; hs-TnT: high-sensitive troponin T; INR: international normalised ratio; *γ*GT: gamma glutamyltransferase.

**Table 2:** Plasma concentrations of AOPPs-albumin and hs-TnT in cirrhotic patients with chronic HCV infection.

	Healthy controls	Child-Pugh	Child-Pugh	Child-Pugh
		A	B	C
(*n*)	40	34	34	20
Age (years)	55 (29–56)	54 (21–74)	58 (24–71)	56 (29–69)
Albumin (g/L)	45 (36–57)	34^*∗*^ (28–45)	30^*∗*^ (20–40)	25^*∗*^ (16–32)
Bilirubin (mg/dL)	0.7 (0.6–0.9)	1.01 (1.02–1.03)	1.56^*∗*^ (1.0–2.0)	2.15^*∗*^ (1.1–3.6)
INR (0.8–1.1)	—	0.9 (0.8–1.09)	1.2 (1.1–1.3)	2.3^+^ (1.6–2.9)
MELD score (6–8)	—	7 (5–10)	9 (8–13)	14^++^ (10–28)
TNF-*α* (pg/mL)	25.0 (20.5–30.2)	34.2 (31.6–45.6)	42.0^*∗*^ (37.6–47.2)	58.7^*∗∗*+^ (48.7–64.0)
hs-CRP (mg/L)	1.05 (0.58–2.5)	3.8^*∗*^ (3.1–7.0)	5.2^*∗∗*^ (4.9–7.7)	6.3^*∗∗*^ (5.8–11.0)
hs-TnT (ng/L)	—	5.2 (3.0–6.6)	5.9 (3.0–8.4)	8.6^+^ (3.0–18.5)
AOPPs-albumin (*μ*mol/g)	1.7 (0.8–2.7)	2.8^*∗*^ (1.3–4.4)	3.2^*∗*^ (1.9–4.5)	4.1^*∗∗*+^ (2.3–5.2)

Continuous variables are expressed as median (interquartile range).

Significance between groups: ^*∗*^ *P* < 0.05; ^*∗∗*^ *P* < 0.01  *v*ersus healthy controls; ^+^ *P* < 0.05; ^++^ *P* < 0.01  *v*ersus Child-Pugh A.

AOPPs: advanced oxidation protein products; hs-TnT: high-sensitive troponin T; INR: international normalized ratio; MELD: model of end-stage liver disease.

**Table 3:** Clinical and biochemical characteristics of patients with liver cirrhosis according to the presence of ascites.

	Healthy controls	No ascites	Ascites
(*n*)	40	35	53
Age (years)	55 (29–56)	51 (21–74)	58 (24–70)
Esophageal varices *n* (%)	—	17 (49)	39 (74)
Refractory ascites *n* (%)	—	—	11 (21)
Child-Pugh score	—	6 (4–7)	9 (8–10)^++^
Creatinine (mg/dL)	0.8 (0.7–1.0)	0.9 (0.7–1.4)	1.32^*∗∗*++^ (0.8–2.4)
Albumin (g/L)	45 (36–57)	34^*∗*^ (28–45)	29^*∗*^ (16–40)
MAP (mmHg)	—	88 (85–93)	77^+^ (73–89)
Plasma renin activity (ng mL^−1^ h^−1^)	—	0.48 (0.13–1.4)	1.9^++^ (0.95–7.6)
Aldosterone (ng/dL)	—	13.2 (5.5–20.9)	33.0^+++^ (13.7–52.2)
Antidiuretic hormone (pg/mL)	—	4.6 (2.5–5.7)	4.5 (3.6–6.4)
TNF-*α* (pg/mL)	25.0 (25.5–30.2)	32.3 (31.6–45.6)	55.0^*∗∗*+^ (37.6–64.0)
hs-CRP (mg/L)	1.05 (0.58–2.5)	3.8^*∗*^ (3.1–7.0)	5.9^*∗∗*^ (4.9–11.0)
hs-TnT (ng/L)	—	5.1 (3.0–6.6)	10.7^++^ (3.0–18.5)
AOPPs-albumin (*μ*mol/g)	1.7 (0.8–2.7)	2.2^*∗*^ (1.3–4.4)	3.6^*∗*+^ (1.9–5.2)

Continuous variables are expressed as median (interquartile range: IQR) and categorical variables as number (percentage). Significance between groups ^+^ *P* < 0.05; ^++^ *P* < 0.01, ^+++^ *P* < 0.001  *versus* cirrhosis without ascites. AOPPs: advanced oxidation protein products; hs-TnT: high-sensitive troponin T; MAP: mean arterial pressure.

**Table 4:** Multiple logistic regression analysis of factors associated with the presence of ascites.

	OR	95% CI	*P* value
AOPPs-albumin level (*μ*mol/g)	0.19	0.21–0.44	0.001
hs-TnT level	0.11	0.11–0.61	0.01
Creatinine level (mg/dL)	0.64	0.02–0.03	0.001

AOPPs: advanced oxidation protein products; hs-TnT: high-sensitive troponin T.
